# A Method of Hyper-sphere Cover in Multidimensional Space for Human Mocap Data Retrieval

**DOI:** 10.2478/v10078-011-0030-0

**Published:** 2011-07-04

**Authors:** Xiaopeng Wei, Boxiang Xiao, Qiang Zhang, Rui Liu

**Affiliations:** 1 Key Laboratory of Advanced Design and Intelligent Computing(Dalian University), Ministry of Education, Dalian, China; 2 School of Mechanical Engineering, Dalian University of Technology, Dalian, China

**Keywords:** MoCap (Motion Capture) Data, Retrieval, Multidimensional Space, Hyper-sphere Cover

## Abstract

A method of hyper-sphere cover in multidimensional space for human Mocap (Motion Capture) data retrieval is presented in this paper. After normalization and feature extraction, both the retrieval instance and the motion data are mapping into a multidimensional space. Several hyper-spheres are constructed according to the retrieval instance, and the domain covered by these hyper-spheres can be considered as the distribution range of a same kind of motions. By use of CMU free motion database, the retrieval algorithm has been implemented and examined and the experimental results are illustrated. At the same time, the main contributions and limitations are discussed.

## Introduction

Human motion capture including motion data processing and retrieval has attracted increasing attention from many researchers in past decades. In the field of sports practice, the human motion capture technique has been widely used for athletes training, motion analysis and so on. With the development and popularity of motion capture device, there is a rapidly growing data of human motion (B. Demuth, et al., 2006). However, the reuse of the motion data is still limited because of the lack of general and efficient motion retrieval systems. The investigation of motion data retrieval approaches is still a hot issue and many works have been developed.

In previous studies, one of developed techniques is motion templates based methods for human motion retrieval and classification. Muller and his colleagues (M. Muller and T. Roder, 2006) proposed a method for automatic classification and retrieval of motion capture data facilitating the identification of logically related motions scattered in some database. Roder introduced templates methods systematically in his doctoral dissertation (T. Roder, 2006). Another technique is content-based or index-based methods. For instance, Chiu et al. (C. Chiu, et al., 2004) put forward to a framework for constructing a content-based human motion retrieval system including two major components: indexing and matching. Muller et al (M. Muller, et al., 2005) presented automated methods for efficient indexing and content-based retrieval of motion capture data. Yamasaki and his colleagues (T. Yamasaki and K. Aizawa, 2007) described a content-based cross search scheme for two kinds of three-dimensional (3D) human motion data: time-varying mesh (TVM) and motion capture data. Chao and colleagues (S. Chao, et al., 2003) presented a simple and effective approach for motion retrieval and synthesis based on posture feature indexing, and posture features of each frame data was extracted by an index function. A 3D motion retrieval method with motion index tree was presented by Liu and colleagues (F. Liu, et al., 2003). Feature analysis is also a common used approach by many researchers. A set of relational motion features was been defined in Demuth’s work (B. Demuth, et al., 2006). Xiang and Zhu (J. Xiang and H. Zhu, 2007) extracted 3D temporal-spatial features of motion data and automatically constructed data driven decision trees. Lin (Y. Lin, 2006) also defined a kind of motion features in his work. Many researchers used dynamic time warping (DTW) for different extent motion sequences (B. Demuth, et al., 2006; C. Chiu, et al., 2004). Furthermore, there are several other methods such as probabilistic principal component analysis (PPCA) proposed by Wang (X. Wang, et al., 2008), movement notation language presented by Yu et al. (T. Yu, et al., 2005), Ensemble HMM Learning based approach developed by Xiang and Zhu (J. Xiang and H. Zhu, 2007), energy morphing based method proposed by Tam et al. (G. Tam, et al., 2007) and semantic matching based method (X. Wei, et al., 2008).

To implement a general and efficient motion retrieval system, we present a retrieval method based on hyper-sphere covering in multidimensional space. To demonstrate the efficiency of this technique, we examine the algorithm by a BVH conversion of CMU free motion database (http://mocap.cs.cmu.edu/). The CMU free motion database is an open human motion database which is constructed by the Graphics Lab of Carnegie Mellon University. The database is composed of 2514 motion sequences which were divided into 6 categories and 23 sub-categories. The BVH which was originally developed by Biovision company, is a file format for Mocap data. The main contribution of this study is that we construct a uniform structure of feature vectors for all motion data which eliminates the differences in geometric parameters. Furthermore, the algorithm is based on the distribution of similar motions in multidimensional space and, and avoids the influences by differences in extent and velocity of motion sequences.

### Overview

**Figure 1:**
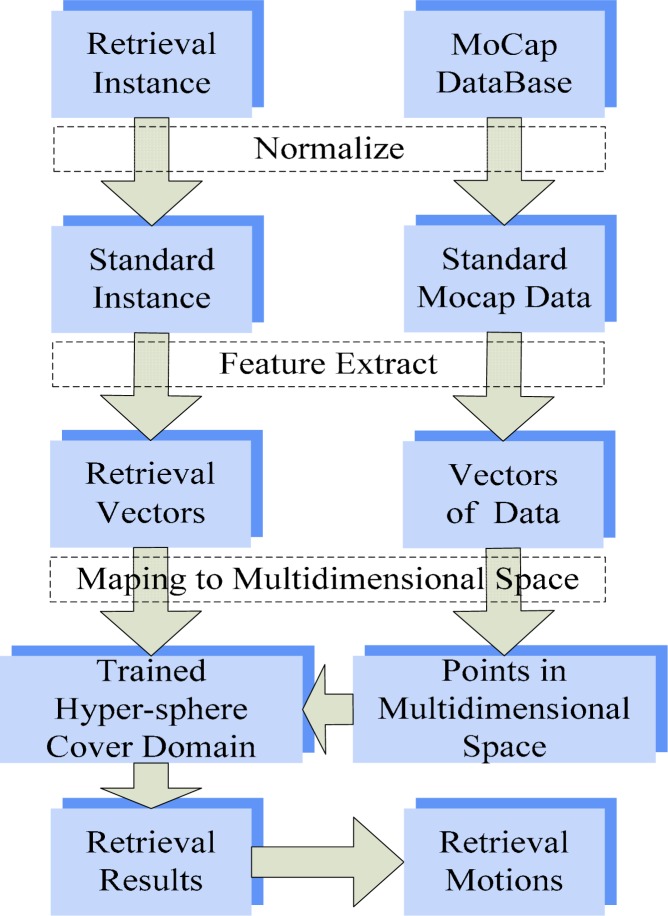
The flowchart of method in this work

The aim of this work is to implement an effective retrieval approach for human motion capture data. The flowchart of method in this paper is shown in Figure 1. In the section of method, our retrieval algorithm is introduced by detail including steps motion normalization, feature extraction, multidimensional space mapping and hyper-sphere covering. Experiment results are based on a subset of a BVH conversion of CMU free motion database, and details are illustrated in Section Results. Finally, in Discussion Section, main advantages and limitations of this method are discussed.

## Methods

### Normalization and Feature Extraction of Motion Data

**Figure 2:**
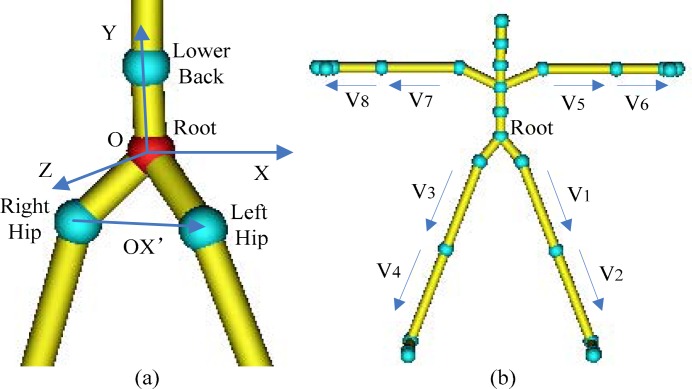
Vectors and the coordinates

Generally, the Mocap data are various and not uniform in coordinates, position, direction or scales, so they must be preprocessed before use. The BVH conversion of CMU motion data is a standard database and all motions are represented by uniform BVH tree and scales, thus the main preprocessing work is to transform the data to a uniform local coordinates. We specify the *Root* joint as the origin of coordinates, note as *O*, and specify the vector from joint *Root* to *LowerBack* as the *OY* axis of coordinates. And then, we choose the vector from joint *RightHip* to *LeftHip* as a preparatory *OX* axis, note as *OX*’, and we can get the *OZ* axis by calculating the orthogonal vector of *OX*’ and *OY*. Finally, we recalculate the true *OX* axis by *OY* and *OZ*. All vectors are normalized and the coordinates are orthogonalized here, shown in Fig 2 (a). We convert each frame of data and retrieval instances to their local coordinates, and all motions and instances are uniformed by accordant coordinates, position, direction and scales.

For the sake of achieving good performance of retrieval algorithm, we choose 8 vectors shown in Fig 2 (b) to define a pose, including upper legs, lower legs, upper arms and lower arms because many motions can be recognized by these main features. Then, we define a 24 dimensional feature vector *M*^24^ *=* (*X*_*V*1_,*Y*_*V*1_,*Z*_*V*1_,*X*_*V*2_, ⋯,*Z*_*V*8_) by the normalized 8 vectors, and all motions and instances are uniformed by accordant coordinates, position, direction and scales.

### Construction of cover domain by Retrieval Instance and Hyper-sphere Covering in Multidimensional Space

**Figure 3:**
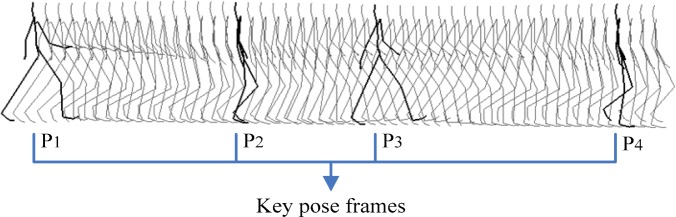
Key poses of walk (heavy black)

The retrieval instance in this work is a short query motion clips. Several frames of key pose in query clips are selected by manual operation, and all key pose frames’ feature vectors mentioned in 3.2 are subsequently extracted. And then, a Remark Sequence of retrieval instance is constructed at the same time. Fig.3 shows an example of one motion clip of walk, in that the 4 heavy black frames are key poses of a walk clip with Remark Sequence[1,2,3,4], and others are general data.

After the extraction of feature vectors of retrieval instance, we construct the cover domain of each kind of pose such as *P_k_* in a multidimensional space by Eq. (1), and the dimension of space is equal to the dimension of feature vectors.

(1) $$\{\begin{array}{c} M_{j,k}^{n}=(X_{1,(j,k)},X_{2,(j,k)},X_{3,(j,k)},\cdots ,X_{n,(j,k)})\;\;\;M_{j,k}^{n}\in R^{n},j\in [1,m],k\in [1,p]\\ S_{j,k}=\left\{Y^{n}|\left\|Y^{n}-M_{j,k}^{n}\right\|\leq \delta \right\}\;\;Y^{n}\in R^{n},j\in [1,m],k\in [1,p]\\ D_{k}=S_{1,k}\cup S_{2,k}\cup S_{3,k}\cup ,\cdots ,\cup S_{m,k},k\in [1,p] \end{array}$$

where:

- $M_{j,p}^{n}$ is one of feature vectors of a pose
- *P_k_*. *n* is the dimension and *n=* 24 here.
- *m* is the number of feature vectors of pose
- *P_k_*, and *p* is the number of key poses in retrieval instance.
- *S_j,k_* is the cover domain of a feature vector of pose
- *P_k_* by a threshold
- δ and *D_k_* is the cover domain of pose *P_k_*.

For motion retrieval, hyper-sphere covering in multidimensional space is to estimate the distribution of every motion data in the space. If a frame’s feature vector in a motion clip belongs to the cover domain *D_k_*, it can be considered as covered pose of *P_k_* and be noted as *k* in Cover Sequence, or else it is noted as 0. Cover Sequence noted as *C* is an integer array to record the cover status of a motion sequence, and for example, it can be simplified by Eq.2. In sequence, zero elements are removed, and continuous same elements are denoted by one element.

(2) $$\begin{array}{c} C=[0,0,0,1,1,0,2,2,2,2,0,0,3,4,4]\\ C_{\mathrm{simple}}=[1,2,3,4] \end{array}$$

The Cover Sequence of a motion sequence is the unique evaluation for retrieval. If a motion’s the Cover Sequence continuously cover the Remark Sequence in order, it is considered as the similar object. Furthermore, part sequence of a sequence is defined to evaluate main similarity, and it is actually the sub sequence of a sequence with one element absent. For example, sequences[1,2,3], [2,3,4], [1,2,4], [1,3,4] are all part sequences of sequence [1,2,3,4].

For instance, if in a Cover Sequence *C_walk_* of a walk sequence, a sub sequence equal to the Remark Sequence of retrieval instance such as[1,2,3,4] in Fig.3 exists, the motion can be considered as the similar object to walk and it can be retrieved. If the Cover Sequence of a motion contains part but whole Remark Sequence of retrieval instance, we can consider it as the main similar object which can also be recalled.

### Experiment conditions and Retrieval accuracy

The motion retrieval system in this work was developed by C++ program language and OpenGL (Open Graphic Library), and experimental results were obtained on a 3.0 GHz Pentium 4 with 2 GB of main memory. We evaluated the system on a subset of BVH conversion of the CMU motion database, which contains 184 motions about 105 thousands frames sampled at 120 Hz of motion capture data including walk, run, jump, cartwheel, swing and their blends.

The retrieval accuracy of the proposed framework is evaluated by the precision and recall, and these accuracy evaluations are adopted by many researchers (C. Chiu, et al., 2004).

$$\begin{array}{c} \text{precision}=\frac{\#\{\text{relevant}\cap \text{retrieved}\}}{\text{retrieved}}\\ \text{recall}=\frac{\#\{\text{relevant}\cap \text{retrieved}\}}{\text{relevant}} \end{array}$$

where #retrieved is the number of retrieved clips and #relevant is the number of relevant clips.

## Results

**Figure 4:**
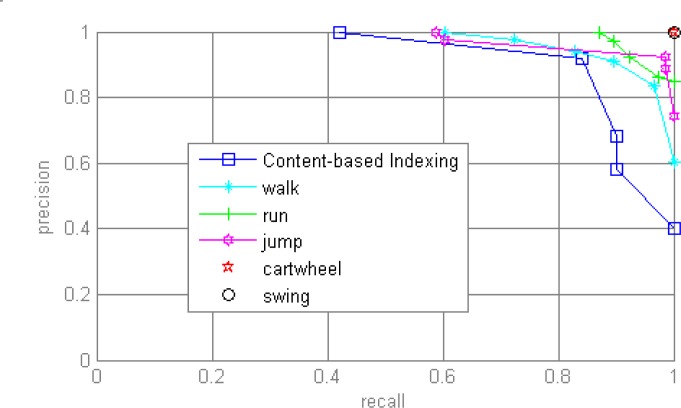
Part results of retrieval accuracy of proposed approach and the comparison to content-based Indexing method

**Figure 5:**
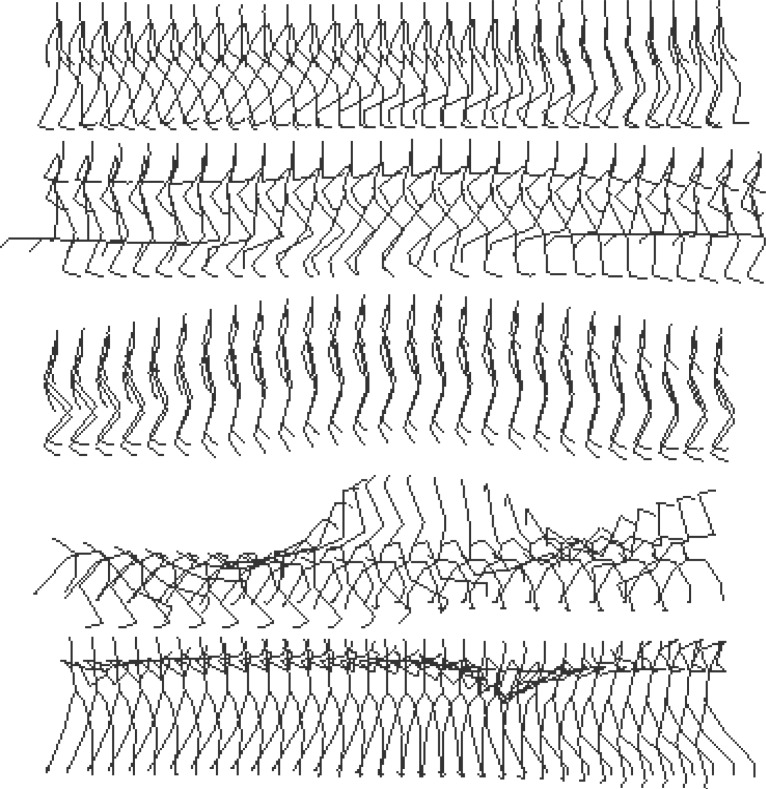
Part visual retrieval results including walk, run, jump, cartwheel, swing from upper to lower

The part results are shown in Fig. 4, and Fig.5 shows part visual retrieval results. In experiments, some motions such as cartwheel and swing achieve results with accuracies at 1.0, because the numbers of samples in database are few respectively at 9 and 10. To evaluate the performance of ours method, we compare our accuracy with the referenced Content-based Indexing method (C. Chiu, et al., 2004), shown in Fig 4.

On the other hand, time cost of approach is evaluated in ours work. The time cost includes two main parts: feature vectors extraction and computation of hyper-sphere covering. A main time-consuming operation is feature vectors extraction for the whole database (about 3 minutes) and the operation needs to be executed only time in all experiments because the structures of feature vectors are uniform for different retrieval instances. Moreover, the time cost of covering computation traversing the whole database about for a retrieval instance is about 22 seconds.

## Discussion

In this paper, we presented a method of hyper-sphere cover in multidimensional space for human Mocap data retrieval. The retrieval instance in this work is a short query motion clips. After normalization and feature extraction, both the retrieval instance and the motion data are mapping into a multidimensional space. By computation of hyper-sphere covering, relevant motions to the retrieval instance are recalled, and experimental results are illustrated.

The advantage of this work is twofold. Firstly, the proposed algorithm is based on the distribution of similar motions in multidimensional space, and avoids the influences by differences in extent and velocity of motion sequences. Furthermore, the normalization of motion data eliminates the differences in geometric parameters including coordinates, position, direction and scales of motions, and the uniform structure of feature vectors ensure the feasibility and efficiency of retrieval in large database.

One limitation of our approach is that some complex freeform motion can not be expressed by distribution in multidimensional space well and it is a difficult task for future work. Moreover, we are also study the dynamic distribution of motions in multidimensional space.
